# A lichenoid drug eruption secondary to pyridostigmine

**DOI:** 10.1016/j.jdcr.2026.04.066

**Published:** 2026-05-12

**Authors:** Cristina Grechin, Tudor Munteanu, Jessica Gale

**Affiliations:** a Dermatology Department, St James’s Hospital, Dublin, Ireland; b Neurology Department, Our Lady of Lourdes Hospital, Drogheda, Ireland

**Keywords:** lichenoid drug eruption, medical education, pyridostigmine

Myasthenia gravis (MG) is a chronic autoimmune disease affecting the neuromuscular junction, leading to debilitating and potentially life-threatening muscular weakness.^1^

MG is also a well-recognized paraneoplastic syndrome associated with thymomas at an estimated incidence of 0.25 to 2 cases per million, reflecting the close interrelation between thymic tissue and MG pathogenesis, as approximately 50% of thymoma patients develop MG and 10% to 20% of MG patients are found to have thymomas.^2^

Pyridostigmine, an acetylcholinesterase inhibitor, is recommended as the first-line treatment for MG.^3^ A number of side effects have been attributed to this medication and pertain mostly to the drug’s amplification of the parasympathetic nervous system. In a cross-sectional study of 410 patients conducted by Remijn-Nelissen et al, 91% of patients using pyridostigmine reported at least 1 side effect.^3^ These most commonly included diarrhoea, flatulence, abdominal cramps, urinary urgency, muscle cramps, blurred vision, hyperhidrosis, increased salivation, light-headedness, and flu-like symptoms.^3^ Fewer than 2% reported skin rash or hives.^3^ Following a literature review, there was only a single reported case of a cutaneous reaction to pyridostigmine, which was a cutaneous vasculitis, presented by Singh et al^4^

We present a case of a lichenoid drug eruption secondary to pyridostigmine.

A 46-year-old man presented to the emergency department with a 1-week history of a progressive, pruritic, erythematous rash affecting his arms, trunk and thighs. The patient had a complex medical history, including recurrent metastatic (liver, lungs, pleural, kidney, bones) thymoma (previously treated with neoadjuvant chemotherapy and radiotherapy, extensive surgical resection), complicated by paraneoplastic MG, bronchiectasis, severe oesophageal achalasia contributing to recurrent aspiration pneumonia and provoked pulmonary embolism.

The rash appeared 2 weeks after initiating pyridostigmine. The patient previously used pyridostigmine 1 year prior to this exposure for approximately 1 week; the reason for discontinuing the treatment at that point was unclear. However, the patient reported nail changes clinically consistent with lichenoid nails a few months after the initial pyridostigmine exposure.

On examination, multiple erythematous macules and patches were observed on the upper chest, along with scattered erythematous macules and papules on the back (Fig 1, *A*). Additionally, there were 4 distinct erythematous patches with a fine collarette of scale on the right flank and 2 similar patches on the right thigh (Fig 1, *B*). Also, there were lichenoid nail changes affecting nails of both hands (Fig 1, *C* and *D*). A skin biopsy from the chest and thigh showed parakeratosis and acanthosis with vacuolar changes, along with necrotic keratinocytes consistent with a drug eruption. Periodic acid-Schiff stains for fungi were negative. The working diagnosis was a grade I drug rash, with pyridostigmine identified as the suspected culprit. The calculated Naranjo score was 5, indicating that pyridostigmine was the probable cause. We prescribed topical betamethasone ointment and emollients. Two weeks later, the pruritus worsened, and the rash continued to progress with more widespread involvement, including the scalp (Fig 2). Moreover, the patient’s quality of life was significantly impacted. Following a discussion with his neurologist, pyridostigmine was discontinued. The potency of the topical steroid was up-titrated to clobetasol propionate once daily. Three months after pyridostigmine was stopped, the rash had completely resolved, with residual mild post-inflammatory hyperpigmentation (Fig 3). A diagnosis of lichenoid drug eruption secondary to pyridostigmine was established.

**Fig 1:**
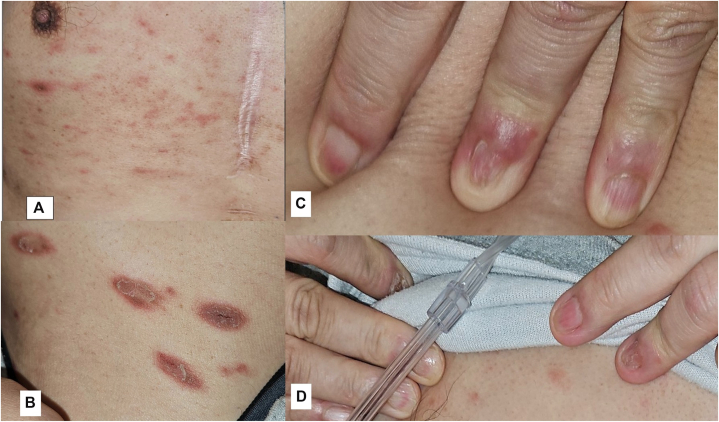
Clinical appearance at rash onset: **(A)** macules and patches of erythema on the trunk, **(B)** 4 distinct erythematous patches with a fine collarette of scale on the right flank, **(C** and **D)** lichenoid nail changes affecting the nails of both hands.

**Fig 2:**
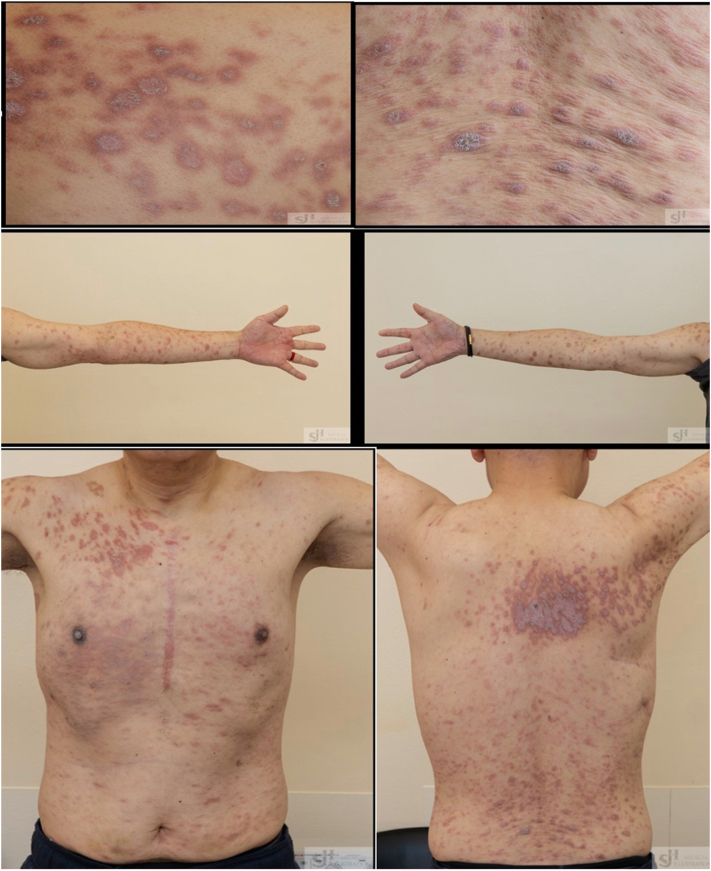
Widespread lichenoid rash with collarettes of scale affecting the whole body at 2 weeks review while still on pyridostigmine.

**Fig 3:**
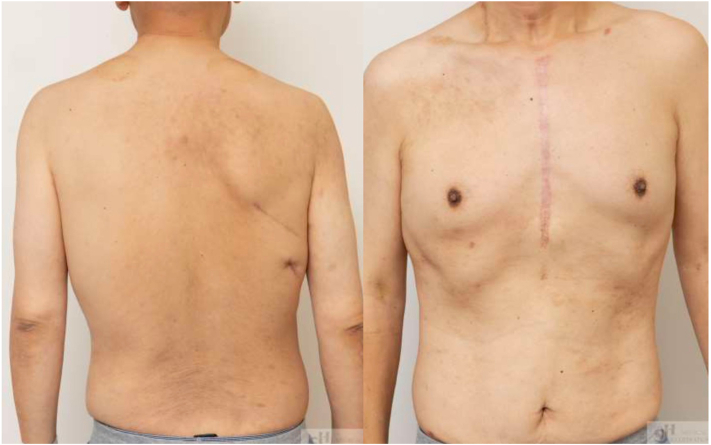
Rash completely resolved 3 months post-pyridostigmine discontinuation.

The patient was subsequently admitted to a different centre with recurrent aspiration pneumonia and generalised weakness. Due to his worsening condition, a decision was made to recommence pyridostigmine and treat with intravenous immunoglobulins (IVIG). The patient died 1 week after recommencing pyridostigmine due to pneumonia. During the week post-exposure, the patient did not develop a rash; however, it is important to highlight that IVIG could contribute to a delay in rash onset in this case.

Cutaneous lichenoid drug eruptions (LDE) are adverse drug reactions characterized by symmetric, erythematous, violaceous papules. They are uncommon adverse drug reactions that share clinical and histological similarities with lichen planus (LP).^5^ Identifying the culprit can be challenging, as symptoms often appear following a significant delay and can resolve slowly, even after discontinuing the offending drug.^5^ LP is recognized as a T-cell-mediated condition where keratinocytes express self-antigens, causing cell death through CD8+ cytotoxic T-cell activation.^5^ Both LP and LDE are associated with high levels of IFN-γ. However, the exact mechanism by which drugs can initiate T-cell activation is not fully understood.^5^

Maul et al conducted a narrative review of cutaneous LDE over a 20-year period.^5^ They reported that checkpoint inhibitors were the most frequently implicated drugs (136 cases; 42.1%), followed by tyrosine kinase inhibitors (39 cases; 12.0%) and anti-TNF-α monoclonal antibodies (13 cases; 4.0%).^5^ Notably, pyridostigmine was not listed among the drugs reported to cause LDE. According to Bhanja et al, other common drugs that cause LDE are anti-hypertensive drugs (enalapril and amlodipine), followed by anti-tubercular drugs (pyrazinamide) and anti-diabetic medications (glimepiride, metformin, and chlorpropamide). However, their study included a single center with a small number of patients.

This report aims to raise awareness among clinicians regarding the association between pyridostigmine and lichenoid eruptions. Further research in this area is warranted.

## Conflicts of interest

None disclosed.
